# A rare cause of nasolacrimal duct obstruction: Dentigerous cyst in the maxillary sinus

**DOI:** 10.4103/0301-4738.57161

**Published:** 2009

**Authors:** Biswarup Ray, Saumendra Nath Bandyopadhyay, Debabrata Das, Bivas Adhikary

**Affiliations:** Department of Ophthalmology and ENT, ^1^Department of Khudiram Bose Sarani, R.G. Kar Medical College & Hospital, Kolkata, India.

**Keywords:** Caldwell Luc approach, dentigerous cyst, nasolacrimal duct

## Abstract

The most common abnormality of the lacrimal drainage system is congenital or acquired nasolacrimal duct obstruction. The causes of acquired nasolacrimal duct obstruction may be primary or secondary. The secondary acquired obstructions may result from infection, inflammation, neoplasm, trauma or mechanical causes. The maxillary sinus cysts usually obstruct the nasolacrimal duct mechanically. Dentigerous cysts are one of the main types of maxillary cysts. These cysts are benign odontogenic cysts which are associated with the crowns of unerupted teeth. The clinical documentations of mechanical nasolacrimal duct obstructions due to a dentigerous cyst in the maxillary sinus are very rare in literature. In this case report, we describe a dentigerous cyst with a supernumerary tooth in the maxillary sinus in an 11-year-old male child causing an obstruction to the nasolacrimal duct. The case was successfully managed surgically by Caldwell Luc approach.

Acquired secondary nasolacrimal duct (NLD) obstruction may result from infection, inflammation, neoplasm, trauma or mechanical causes.[1] The mechanical obstruction of NLD by a dentigerous cyst in the maxillary sinus has been rarely documented in literature.[2,3] NLD obstruction presents commonly with epiphora and acute or chronic dacryocystitis.[2] We herein report this unusual case which presented to us with epiphora due to complete NLD obstruction by a dentigerous cyst containing a supernumerary tooth in the maxillary sinus.

## Case Report

**Figure 1:**
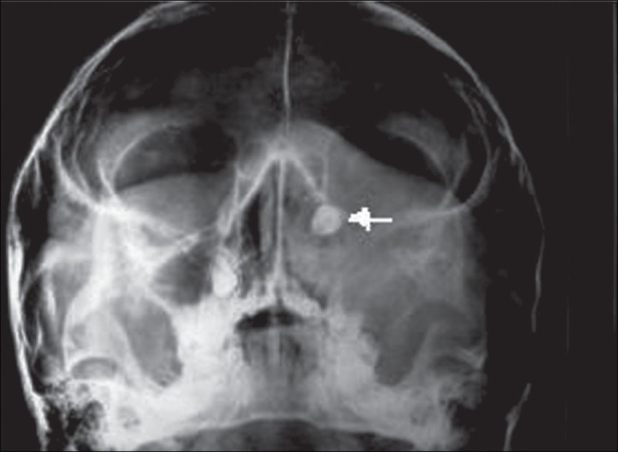
X-ray of the paranasal sinuses showing haziness of the left maxillary antrum with the dental structure near its roof (white arrow)

**Figure 2:**
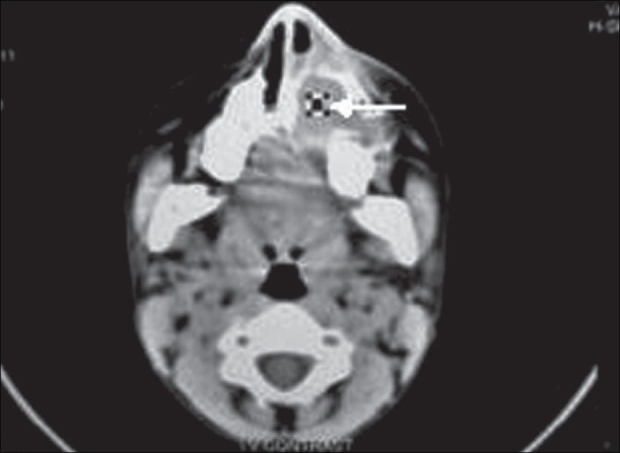
Axial CT scan of the orbits and sinuses showing the cyst bulging into the nasal cavity and the tooth in the left maxillary antrum (white arrow)

**Figure 3:**
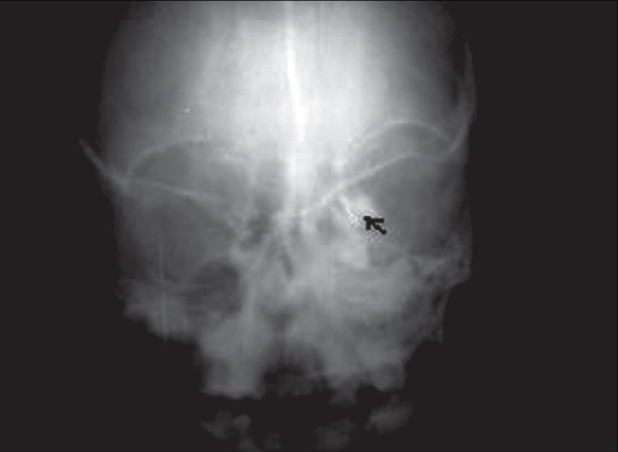
Preoperative left dacryocystogram showing inability of the contrast medium to pass beyond the lacrimal sac (black arrow)

**Figure 4:**
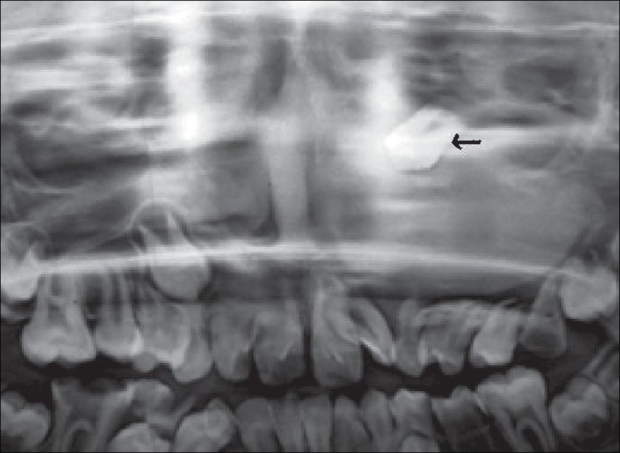
Orthopantogram showing a supernumerary tooth in the left maxillary antrum (black arrow)

**Figure 5:**
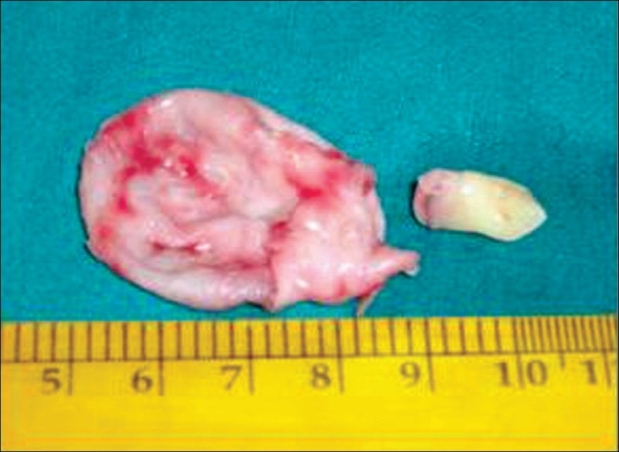
The operative specimen of the cyst and the tooth

**Figure 6:**
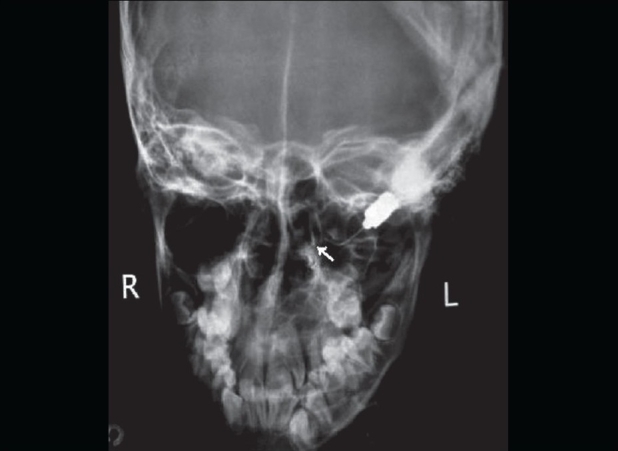
Postoperative left dacryocystogram showing flow of the contrast medium into the nose (white arrow)

An 11-year-old male child was referred to us for epiphora. He had intermittent mucoid discharge from the inner canthus of the left eye for the last one year. Ocular and medical histories were normal. On examination, his best-corrected visual acuity was 20/20 in both eyes. Pupillary reflexes and extraocular movements were normal. Intraocular pressures were 16 mm of Hg in both eyes. Slit-lamp and funduscopic examinations were normal. On compression of the left lacrimal sac mucoid material regurgitated from the puncta. Lacrimal sac syringing revealed regurgitation of fluid from the opposite punctum and hard stop with probing, suggestive of NLD obstruction. Radiograph of the paranasal sinuses showed opacified left maxillary antrum with a retained tooth near its roof [Fig. 1]. A plain computed tomography (CT) scan of the orbits and paranasal sinuses was done by taking 2-mm axial and 3-mm coronal slices. The CT images were suggestive of a maxillary sinus cyst close to the orbital floor with the crown of the affected tooth projecting into it [Fig. 2]. The cystic mass was pushing the naso-antral wall into the nasal cavity and compressing the NLD. There was no compression of the globe. Left dacryocystogram showed inability of the contrast medium to pass beyond the lacrimal sac [Fig. 3]. Orthopantogram (OPG) detected the presence of a supernumerary tooth in the left maxillary antrum [Fig. 4]. Our colleagues of the ENT department were consulted for management of the cyst in the maxillary sinus. Their examination revealed congested left nasal cavity with grossly restricted airway and bulging of the lateral wall of the left nose. There was no missing tooth. Removal of the cyst was planned by Caldwell Luc's approach. The outer part of the antero-lateral wall of maxilla was thin and its removal exposed the tense cyst wall. About 8 ml of purulent material was aspirated from the cyst. The cyst was removed from the maxillary sinus along with the retained tooth [Fig. 5]. The maxillary sinus walls were found to be intact. Aspirated material on culture was found to be sterile. The postoperative course was uneventful and his symptoms resolved promptly. Postoperative left dacryocystogram revealed free flow of the contrast medium into the nose [Fig. 6]. On six months follow-up the child remained asymptomatic.

## Discussion

Nasolacrimal duct (NLD) obstruction may occur due to congenital or acquired causes. Acquired obstruction may be primary or secondary, complete or partial. Primary obstructions are idiopathic and secondary obstructions may be due to infection, inflammation, neoplasm traumatic or mechanical causes.[1] Mechanically, a maxillary sinus cyst can obstruct NLD. The different types of maxillary cysts are antral mucoceles, retention cysts, pseudocysts and dentigerous cysts.[3] Dentigerous cysts are usually solitary, benign odontogenic cysts associated with the crowns of unerupted teeth.[4] The accumulation of fluid between the unerupted tooth and the surrounding dental follicle is the accepted etiology of the cyst. Third molars followed by maxillary canines (the most commonly impacted teeth) and only occasionally supernumerary teeth or odontomas are involved in cyst formation.[5] Supernumerary or ectopic tooth in the maxillary sinus is rare.[2,6] The common presentations of dentigerous cysts are facial swelling, delayed tooth eruption, recurrent head and neck infections.

As the cyst enlarges it expands the maxillary sinus and presses over the adjacent structures. In our case the dentigerous cyst was compressing the NLD. The easiest way to remove a dentigerous cyst is through a Caldwell Luc approach.[7] In this approach a window of adequate size is created on the anterior wall of the maxilla to deliver the cyst along with the impacted tooth. Though CT scan differentiates between a cyst and a tumor, aspiration with a 16/18-gauge needle can be done for confirmation.[8] Enucleation of the cyst usually results in complete resolution of symptoms without evidence of recurrence.[2,9] If the obstruction persists after surgery for maxillary cyst, a dacryocystorhinostomy is needed as a bypass procedure.[3]

Despite the possibility of dentigerous cysts of maxillary sinus causing NLD obstruction, after extensive MEDLINE search, we observed that only four cases have been reported so far.[2,3,9] In 1997 Altas *et al*.[9] reported a case of a large dentigerous cyst with a canine tooth in the right maxillary antrum causing NLD obstruction. In 2000 Alexandrakis *et al*.[2] reported two cases of NLD obstruction by an ectopic maxillary sinus and intranasal tooth. In 2003 Bajaj *et al*.[3] reported a case of dentigerous cyst in the maxillary sinus with NLD obstruction.

We conclude that the possibility of ectopic eruption of teeth should be kept in mind while evaluating a case of secondary NLD obstruction.

## References

[1] Bartley GB (1992). Acquired lacrimal drainage obstruction: An etiologic classification system, case reports, and a review of the literature. Ophthal Plast Reconstr Surg.

[2] Alexandrakis G, Hubbell RN, Aitken PA (2000). Nasolacrimal duct obstruction secondary to ectopic teeth. Ophthalmology.

[3] Bajaj MS, Mahindrakar A, Pushker N (2003). Dentigerous cyst in the maxillary sinus: A rare cause of nasolacrimal obstruction. Orbit.

[4] Micozkadioglu SD, Erkan AN (2007). Endoscopic removal of a maxillary dentigerous cyst. B-ENT.

[5] Motamedi MHK, Talesh KT (2005). Management of extensive dentigerous cysts. British Dental Journal.

[6] Bodner L, Tobi F, Bar-Ziv J (1997). Teeth in the maxillary sinus: imaging and management. Journal of Laryngology and Otology.

[7] Manzoor T, Raza SN, Qayyum A, Azam K (2006). Dentigerous cyst presenting as facial pain. J Coll Physicians Surg Pak.

[8] Miller RH, Sturgis EM, Sutton CL, Ballenger JJ, Snow JB (1996). Neoplasms of the nose and paranasal sinuses. Otorhinolaryngology: Head and neck surgery.

[9] Atlas E, Karasen RM, Yilmamaz AB (1997). A case of large dentigerous cyst containing a canine tooth in the maxillary antrum leading to epiphora. Journal of Laryngology and Otology.

